# *Laurus nobilis*, *Salvia sclarea* and *Salvia officinalis* Essential Oils and Hydrolates: Evaluation of Liquid and Vapor Phase Chemical Composition and Biological Activities

**DOI:** 10.3390/plants10040707

**Published:** 2021-04-06

**Authors:** Elisa Ovidi, Valentina Laghezza Masci, Marta Zambelli, Antonio Tiezzi, Sara Vitalini, Stefania Garzoli

**Affiliations:** 1 Department for the Innovation in Biological, Agrofood and Forestal Systems, Tuscia University, 01100 Viterbo, Italy; eovidi@unitus.it (E.O.); laghezzamasci@unitus.it (V.L.M.); marta.zambelli1995@gmail.com (M.Z.); antoniot@unitus.it (A.T.); 2 Department of Agricultural and Environmental Sciences, University of Milan, 20122 Milano, Italy; sara.vitalini@unimi.it; 3 Department of Drug Chemistry and Technology, Sapienza University, 00185 Rome, Italy

**Keywords:** officinalis plants, essential oils, hydrolates, HS-GC/MS, chemical composition, antibacterial activity, antioxidant activity

## Abstract

*Laurus nobilis*, *Salvia officinalis* and *Salvia sclarea* essential oils (EOs) and hydrolates (HYs) were investigated to define their chemical compositions and biological properties. Gas-chromatography/Mass-spectrometry (GC/MS) and Headspace-GC/MS (HS-GC/MS) techniques were used to characterize the liquid and vapor phase chemical composition of EOs and HYs. 1,8-Cineole (42.2%, 33.5%) and α-pinene (16.7%, 39.0%) were the main compounds of *L. nobilis* EO; 1,8-cineole (30.3%, 48.4%) and camphor (17.1%, 8.7%) were for *S. officinalis* EO; linalyl acetate (62.6%, 30.1%) and linalool (11.1%, 28.9%) were for *S. sclarea* EO for the liquid and vapor phase, respectively. Chemical profile of HYs was characterized by 1,8-cineole (65.1%, 61.4%) as a main constituent of *L. nobilis* and *S. officinalis* HYs, while linalool (89.5%) was the main constituent of *S. sclarea* HY. The antioxidant activity of EOs and HYs was carried out by DPPH and ABTS assays and antimicrobial properties were also investigated by microdilution and the disc diffusion method for liquid and vapor phase against five different bacterial strains such as *Escherichia coli* ATCC 25922, *Pseudomonas fluorescens* ATCC 13525 and *Acinetobacter bohemicus* DSM 102855 among Gram-negative and *Bacillus cereus* ATCC 10876 and *Kocuria marina* DSM 16420 among Gram-positive. *L. nobilis* and *S. officinalis* EOs demonstrated considerable antibacterial activity, while *S. sclarea* EO proved to be less effective. Agar diffusion method and vapor phase test showed the EOs activity with the biggest halo inhibition diameters against *A. bohemicus* and *B. cereus*. A remarkably high antioxidant activity was determined for *L. nobilis* showing low EC_50_ values and also for *S. sclarea;* good EO results were obtained in both of the used assays. *S. officinalis* EC_50_ values were slightly higher to which corresponds to a lower antioxidant activity. Concerning the HYs, the EC_50_ values for *L. nobilis*, *S. officinalis* and *S. sclarea* were remarkably high corresponding to an extremely low antioxidant activity, as also obtained by expressing the values in Trolox equivalent antioxidant capacity (TEAC).

## 1. Introduction

Medicinal and aromatic plants, characterized by a particular aroma and flavor, are among the first remedies that humans used to treat diseases and are a source of biologically active molecules [1,2,3]. Essential oils (EOs) are produced by the plant secondary metabolism and mainly obtained by the steam distillation process [4]. In the EOs isolation process, Hydrolates (HYs) are also obtained as products of the steam distillation, and a small amount of EO constituents dissolve in HYs. Precious oxygenated compounds, which provide specific organoleptic properties and flavor, as well as biological activity, make them useful for food and cosmetic industries [5,6].

*Laurus nobilis*, known as laurel, is a perennial shrub which is widespread in temperate and warm regions, especially in the Mediterranean area where is cultivated as an ornamental plant reaching considerable size [7]; it grows spontaneously in the environment and is a diagnostic taxon of many habitats such as Lauro nobilis–Sambucion nigrae, Lauro nobilis-Tilion platyphylli, and Lauro nobilis–Ulmion minoris [8,9]. Laurel belongs to the Lauraceae family, which comprises approximately 2500 species, and was well-known in antiquity for being a symbol of peace and victory in Greece and ancient Rome, both in the military and in sport competitions, with its intertwined branches to create crowns. The use of laurel in foods is nowadays diffused and its particularly odorous leaves and berries are added to various dish preparations. In previous studies, the chemical composition of liquid phase *L. nobilis* EO was investigated and 1,8-cineole, α-terpinyl acetate, α-pinene and α-terpineol resulted among the main components [10,11,12]. During time laurel gained a considerable importance in traditional medicine [13] and its leaf extracts or EO were used as gastroprotective and antidiarrheal [14,15], analgesic and anti-inflammatory [16], as antidiabetic and to decrease the risk factors of cardiovascular diseases [17,18]. The cytotoxic, antibacterial and antioxidant activities were also investigated, and different authors reported anticancer, antioxidant, insecticidal and molluscidal, antidiabetic, antimicrobial and antifungal activities by *L. nobilis* [19].

Among the Lamiaceae family, *Salvia* genus, whose name comes from the Latin “salvare” which means “to heal, to cure,” has always been considered to possess numerous medicinal properties in traditional medicine of Asia and the Middle East. About 1000 species belong to this genus and they are herbaceous, suffruticosus or shrubby perennial plants displaying a remarkable diversity in growth forms, in floral morphology and pollination biology, and in secondary metabolites production [20]. The chemical composition of Salvia species is particularly rich in sesquiterpenoids, diterpenoids, triterpenoids, steroids, polyphenols and others [21,22], and bioactive molecules were reported to exert proapototic and anticancer activities, to act in prevention and treatment of cardiovascular diseases, to be therapeutic in liver diseases, to be active in the central nervous system as a potential cognitive protective, and to prevent or disaggregate beta amyloid and fibrils as inhibitors of acetylcholinesterase [23,24,25,26,27,28,29,30,31].

To our knowledge this is the first study on the liquid and vapor phase chemical characterization of *L. nobilis, S. officinalis* and *S. sclarea* EOs and their HYs carried out by the headspace-gas-chromatography/mass-spectrometry (HS-GC/MS) technique. The antibacterial activity of EOs and HYs was also investigated by microdilution and disc diffusion methods; furthermore, DPPH and ABTS assays were performed to evaluate their antioxidant activities.

## 2. Results

### 2.1. Liquid and Vapor Phase EOs Chemical Composition

Liquid and vapor phase chemical composition of *L. nobilis*, *S. officinalis* and *S. sclarea* was described by Gas Chromatography-Mass Spectrometry (GC/MS) and Head-Space (HS)-GC/MS analysis. All components identified in *L. nobilis* EO (26 in the liquid phase and 14 in the vapor phase) are reported in Table 1. There was a prevalence of monoterpenes over sesquiterpenes which were missing in the vapor phase. In particular, 1,8-cineole was the most abundant compound (42.2%, 33.5%) followed by α-pinene (16.7%, 39.0%) and β-pinene (13.6%, 10.9%) in the liquid and vapor phase, respectively.

Twenty-three compounds were identified in the liquid phase and twelve in the vapor phase of *S. officinalis* EO and they are listed in Table 2. The chemical composition of EO was dominated by oxygenated monoterpenes (67.7% and 68.4%) followed monoterpenes hydrocarbons (19.1% and 31.4%) in the liquid and vapor phase, respectively. The main constituents of both phases were 1,8-cineole (30.3%, 48.4%), camphor (17.1%, 8.7%), α-thujone (9.7%, 7.0%), camphene (7.9%, 2.2%) and chrysanthenone (6.8%, 3.9%). β-Caryophyllene (0.1%) was the only sesquiterpene also detected in the vapor phase.

*S. sclarea* EO chemical composition is reported in Table 3. Fifteen and thirteen compounds were identified in the liquid and vapor phase, respectively. Oxygenated monoterpenes were the most abundant compounds (76.6% and 62.9%), among which linalyl acetate reached the higher percentages in both phases (62.6% and 30.1%) followed by linalool (11.1%, 28.9%). β-Copaene (6.7%) and β-cubebene (5.0%) were the main sesquiterpene hydrocarbons detected in the liquid and vapor phase EO, respectively.

### 2.2. Vapor Phase HYs Chemical Composition

**Figure 1:**
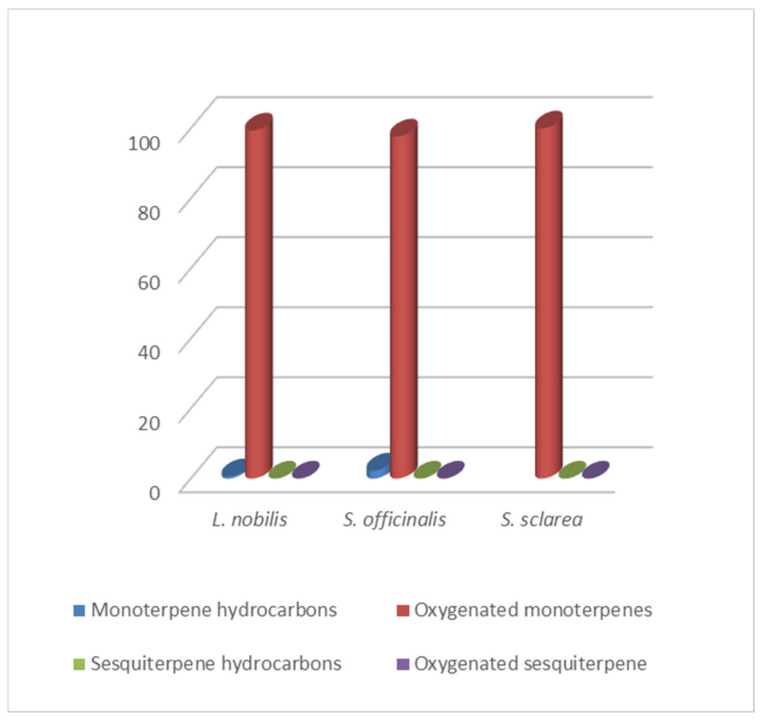
Bar graph of terpene hydrocarbons percentage trend in HYs.

**Figure 2:**
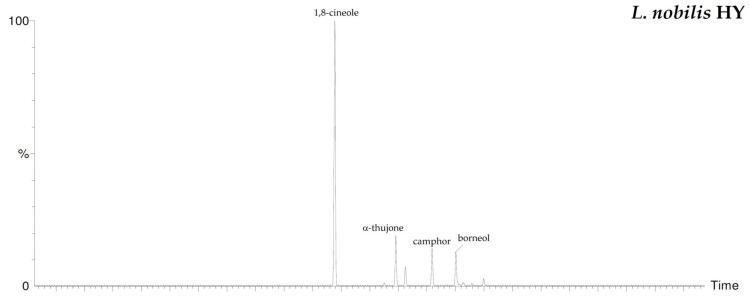
HS/GC-FID chromatograms of *L. nobilis* HY.

**Figure 3:**
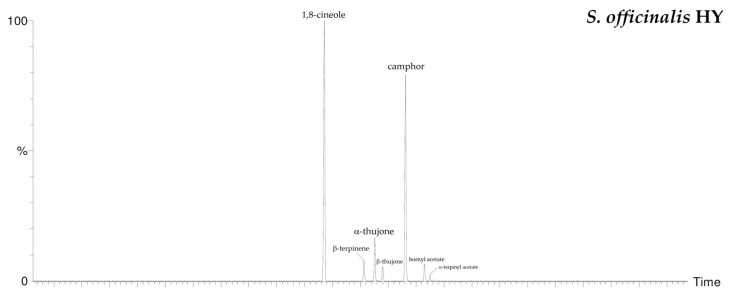
HS/GC-FID chromatograms of *S. officinal*is HY.

**Figure 4:**
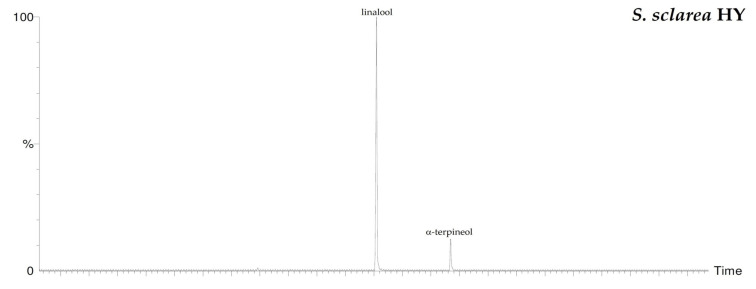
HS/GC-FID chromatograms of *S. sclarea* HY.

By HS-GC/MS analysis, the composition of the vapor phase of all HYs was described. Seven compounds were identified in *L. nobilis* and in *S. officinalis* and only two in *S. sclarea*, and their percentage values are reported in Table 4. 1,8-Cineole (65.1%, 61.4%) was the main compound in both *L. nobilis* and *S. officinalis* HYs, followed by α-thujone (11.1%) and camphor (22.5%) in the first and second HY, respectively. On the other hand, linalool (89.5%) and α-terpineol (10.5%) were the only two compounds detected in *S. sclarea* HY. Sesquiterpenes compounds were missing in all the HYs (Figure 1). The chromatograms of HYs analyses were also reported (Figure 2, Figure 3 and Figure 4).

### 2.3. Antibacterial Activity of L. nobilis, S. officinalis and S. sclarea EOs and HYs

To define the bactericidal and inhibitory activities of the investigated aromatic plants, microdilution assay, expressed as MIC (Minimum Inhibitory Concentration) and MBC (Minimal Bactericidal Concentration) and agar diffusion method and vapor phase test expressed as mm of inhibition diameter of the halo for the liquid (IZ) and vapor phase (VIZ), respectively, were carried out. Furthermore MBC/MIC ratios, which consider an agent as bacteriostatic when the MBC/MIC ratio > 4 and as bactericidal when the MBC/MIC ratio ≤ 4 [32], were reported. In Table 5, *L. nobilis* antibacterial activity against the tested bacterial strains is listed. EO showed MIC and MBC values of 3.13% against *E. coli* and *P. fluorescens*, MIC value of 0.78% and MBC value of 1.56% against *A. bohemicus*, MIC value of 1.56% and MBC value of 6.25% against *K. marina*, and MIC and MBC values of 1.56% against *B. cereus*. For all bacterial trains, MBC/MIC ratios were < 4, confirming a bactericidal activity. Concerning the inhibition halos obtained by the agar diffusion method and by the vapor phase test, the EO IZs were 18.67 ± 2.31 mm against *E. coli* and 7.33 ± 0.58 mm against *A. bohemicus* while, concerning the corresponding EO VIZ, no antibacterial activity was observed for both strains. On the contrary, EO activities against *A. bohemicus*, *K. marina* and *B. cereus* were observed for both liquid and vapor phase with the following inhibition halos diameter: 17.67 ± 2.31 mm, 24.67 ± 3.21 mm and 37.67 ± 2.08 mm for IZs, respectively and 45.67 ± 4.04 mm, 26.67 ± 2.52 mm and 47.33 ± 2.52 mm for VIZs, respectively. No activity was observed for *L. nobilis* HY in the microdilution assay, agar diffusion method and vapor phase test.

In Table 6, the treatment with *S. officinalis* EO showed MIC and MBC values of 6.25% for *E. coli* and *P. fluorescens*, while MIC and MBC values were lower (0.39% and 0.78%, respectively) for *A. bohemicus.* The antibacterial activity against *K. marina* was 1.56% and 0.78% against *B. cereus* for both MIC and MBC values, respectively. MBC/MIC ratios defined the *S. officinalis* EO as bactericidal against all bacterial strains (MBC/MIC < 4). An agar diffusion method showed IZ diameters for liquid phase of 16.67 ± 1.53 mm, 8.00 ± 1.00 mm, 13.67 ± 1.53 mm, 38.33 ± 2.89 mm and 24.33 ± 3.06 mm against *E. coli*, *P. fluorescens*, *A. bohemicus, K. marina* and *B. cereus*, respectively. In the vapor phase test *S. officinalis* EO was active against *A. bohemicus*, *K. marina* and *B. cereus* with 21.67 ± 1.53 mm, 26.67 ± 1.15 mm and 23.00 ± 1.53 mm, respectively; null activity was found against *E. coli* and *P. fluorescens*. As for *L. nobilis* EO, *S. officinalis* HYs were not active against the tested bacterial strains.

The results for *S. sclarea* EO and HYs antibacterial activities are reported in Table 7. MIC and MBC values for the EO were 12.50%, 1.56%, 6.25%, and 6.25% for *E. coli*, *P. fluorescens*, *K. marina* and *B. cereus,* respectively. MBC/MIC ratios defined the *S. sclarea* EO as bactericidal against the sensible bacterial strains. *A. bohemicus* was not affected by the *S. sclarea* EO treatment. Inhibition halos were present in the treatment with *S. sclarea* EO against *A. bohemicus*, *B. cereus* and *K. marina* with 12.67 ± 2.52 mm, 18.67 ± 0.58 mm and 10.67 ± 1.15 mm for liquid phase, while *E. coli* and *A. bohemicus* results were not affected. In the vapor phase test, *S. sclarea* EO did not inhibit the bacterial growth for all the tested strains, and both the liquid and the vapor phase HY were not active.

### 2.4. Antioxidant Activity of L. nobilis, S. officinalis and S. sclarea for EOs and HYs

Chemical assays were used to determine the ability of the tested EOs and HYs to act as a scavenger of free radicals evaluating the decay of 2,2-diphenyl-1-picrylhydrazyl (DPPH) and 2,2′-azinobis-(3-ethylbenzothiazoline-6-sulfonic acid) (ABTS+•) radicals. *L. nobilis*, *S. officinalis* and *S. sclarea* EOs antioxidant activity and that of the corresponding HYs are reported in Table 8, Table 9 and Table 10. In both performed tests—DPPH and ABTS assays—*L. nobilis* essential oil showed the highest antioxidant activity with low IC_50_ values (0.18 ± 0.04 µg/mL and 2.58 ± 0.08 µg/mL, respectively), while higher IC_50_ values (218.10 ± 29.60 µg/mL and 391.38 ± 8.72 µg/mL, respectively) were reported for its HY. Confirming this, TEAC values (Trolox equivalent antioxidant capacity) for *L. nobilis* EO were high (92.97 ± 6.76 mol/mg and 158.49 ± 5.15 mol/mg) for DPPH and ABTS assays, respectively, while the HY exhibited lower values (0.08 ± 0.01 mol/mg and 1.19 ± 0.21 mol/mg, respectively) (Table 8).

The radical scavenging activity for *S. officinalis* EO was calculated and IC_50_ values were 14.10 ± 0.17 µg/mL and 43.64 ± 2.51 µg/mL, while the corresponding HY showed higher values (135.58 ± 33.32 µg/mL and 551.38 ± 17.33 µg/mL) for DPPH and ABTS assays, respectively. Furthermore, *S. officinalis* EO showed TEAC values of 1.28 ± 0.00 mol/mg and 9.26 ± 0.55 mol/mg, while its HY showed TEAC values of 0.14 ± 0.03 mol/mg and 0.76 ± 0.02 mol/mg for DPPH and ABTS assays, respectively (Table 9).

In regards to *S. sclarea*, EO displayed a high antioxidant activity resulting in the IC_50_ values of 7.79 ± 1.06 µg/mL and 2.26 ± 0.05 µg/mL in DPPH and ABTS, respectively, while the corresponding values for the HY were 200.43 ± 28.46 µg/mL and 479.27 ± 7.89 µg/mL. EO showed TEAC values of 2.34 ± 2.36 mol/mg and 186.23 ± 4.30 mol/mg, while the corresponding HY showed values of 0.09 ± 0.01 mol/mg and 0.87 ± 0.01 mol/mg in DPPH and ABTS assays, respectively (Table 10).

## 3. Discussion

GC/MS and HS-GC/MS techniques were carried out to describe the chemical composition of EOs (liquid and vapor phase) and HYs. In particular, using the headspace autosampler, as carried out in this case, no solvent extraction process is necessary for the analysis of HYs. The chemical characterization of *L. nobilis* EO showed a high eucalyptol content for both the liquid and vapor phase (30.4% and 48.4%) as well as in the HY (65.1%), and these data agreed with the reported values [33,34,35].

In our work, 1,8-cineole was the main detected component in the liquid (30.4%) and vapor (48.4%) phase of *S. officinalis* EO and HY (61.4%). *S. officinalis* EOs collected from north of Tunisia, Hatay (Turkey) and Albania showed 1,8-cineole (33.27%, 60.72% and 26.9%, respectively) as the main component [36,37,38]. On the contrary, Baydar et al. [39] reported α-thujone (20.06%) and camphor (43.38%) as the major compounds of EO and HY, respectively, in plants from Isparta, Turkey. α-Thujone was also the principal constituent in EOs from Mexico and California (18.8% and 27.4%) [38], while in two Italian sites eucalyptol (from 40.22% to 60.94%) was the chemical compound characterizing the related wild *Salvia fruticosa* subsp. *thomasii* [22].

Chemical volatile composition of *S. sclarea* EO was characterized by linalyl acetate as the major compound (62.6% liquid phase, 30.1% vapor phase), while HY was characterized by linalool (89.5%). Percentage values of linalyl acetate and linalool in *S. sclarea* EOs vary according to the geographical origin: Serbia (52.83%; 18.18%) and Turkey (11.30%; 8.50%), respectively [40,41], whereas linalyl acetate reached 34.89% and 52.7% and linalool reached 28.91% and 25.65% (inflorescences at full-flowering and at the beginning of seed ripeness stages, respectively) in *S. sclarea* EO from Sicily (Italy) [42].

Variations in the chemical composition of EOs depend on many factors such as the quality of the plant material, the part of the plant used for extraction, the characteristics of the climate and soil, the harvest time, as well as methods and times extraction used for the production and analysis [39,43,44,45,46,47]. Depending on the distillation time and method, the chemical profile of HYs can also vary [48].

Our biological assays, the microwell dilution method, agar diffusion method and vapor phase test, showed antibacterial activity of the investigated aromatic plants against the tested bacterial strains. *L. nobilis* EO showed a high antibacterial activity with MIC values ranging from 0.78% to 3.13% and MBC values from 1.56% to 3.13%. For all bacterial strains the IZ halos were detected (from 37.67 ± 2.08 mm to 7.33 ± 0.58 mm) and the vapor phase proved to be even more active against *A. bohemicus* (VIZ 45.67 ± 4.04 mm), *K. marina* (VIZ 26.67 ± 2.52 mm) and *B. cereus* (VIZ 47.33 ± 2.52 mm). No VIZs were detected against the *E. coli* and *P. fluorescens*.

Concerning *S. officinalis* EO, the obtained results confirmed the antibacterial properties of the EO, showing MIC values from 0.39% to 6.25%, MBC values from 0.78% to 6.25% and IZ values from 38.33 ± mm to 8.00 ± mm. As for *L. nobilis*, a high antibacterial activity for the EO vapor phase was recorded against *A. bohemicus, K. marina* and *B. cereus* with the inhibition halos of 21.67 ± 1.53 mm, 26.67 ± 1.15 mm and 23.00 ± 3.61 mm; *E. coli* and *P. fluorescens* were not affected by the treatment.

Interestingly, the larger inhibition halos were obtained in *L. nobilis* EO against *A. bohemicus* and *B. cereus* in vapor phase tests and in *S. officinalis* EO against *K. marina,* both in the liquid and vapor phase. The agar diffusion test showed activities for *L. nobilis* EO against *E. coli* and *P. fluorescens* with a smaller diameter.

These findings highlighted that *L. nobilis* and *S. officinalis* EOs were active against *E. coli* and *P. fluorescens* growth; Gram-negative bacteria, usually more resistant than Gram-positive for the presence of a complex outer membrane rich in lipopolysaccharide, does not allow the diffusion of hydrophobic molecules present in the essential oils [49]; however, some exceptions were reported [50,51].

*S. sclarea* EO was less effective, showing MIC and MBC values from 12.5% and 1.56%, and no activity was reported against *P. fluorescens*. The agar diffusion test revealed that the EO was active against *A. bohemicus*, *K. marina* and *B. cereus* with smaller IZ halos. On the contrary, the vapor phase test for *S. sclarea* revealed no activity for all bacterial strains.

On the other hand, the investigated aromatic plant HYs were not active against the bacterial strains at tested concentrations. However, it is known that HYs have a lower biological activity than the corresponding essential oils [52] and their potentially antimicrobial activity is also significantly influenced by the distillation method used [33].

As reviewed by Alejo-Armijo et al. [19], *L. nobilis* possess numerous biological properties and the antimicrobial activities were demonstrated against a broad spectrum of human pathogenic microorganisms, pathogens and spoilage bacteria associated with food [37,53,54]. Its antimicrobial activity is probably related to the high percentage of 1,8-cineole (42.2%) and α-pinene (39.0%) detected in the liquid and vapor phase. 1,8-Cineole was found to be active against the development of biofilms formed by the methicillin-resistant strains [55], and its antibacterial activity is enhanced when combined with carvacrol [56]. α-Pinene also demonstrated antibacterial activity [57].

In the present investigation, *S. officinalis* EO was also characterized by 1,8 cineole (30.4% and 48.4%) as the most abundant compound both in the liquid and in vapor phase, while *S. sclarea* EO, which was less active among the aromatic plants investigated, was rich in linalyl acetate (62.6%) and linalool (28.9%) in the liquid and vapor phase, respectively.

The chemical composition of different aromatic and medicinal herb EOs and their antimicrobial activities against human pathogenic bacteria were studied and a scale of antibacterial activities of their main compounds was defined; the linalyl acetate and linalool were active to a minor extent [58,59]. The liquid and the vapor phase antibacterial activity reported in this study for *L. nobilis*, *S. officinalis* and *S. sclarea* EOs was probably due to the synergistic action of their constituent compounds. In this regard, we assumed that the observed antibacterial action may be explained by the synergistic effect of the different components and/or by the presence of other active molecules, even in small amounts.

The development of antibiotic resistant strains caused the failure of antibiotic therapies with serious consequences on animal and human health. The use of EOs as antibacterial agents opens the prospects for new molecules and new strategies to overcome the infectious; similarly, in crop production the use of such compounds could be an alternative to chemicals against various bacteria and fungi [60,61]. In this view, deeper considerations should be given regarding the EOs mechanism of action based on long-term exposure to pathogens and the synergistic effects for the use of EOs-based antibacterial complexes [62].

In regards to the antioxidant activity, *L. nobilis* EO was characterized by low EC_50_ values, 0.18 ± 0.04 µg/mL and 2.58 ± 0.08 µg/mL (DPPH and ABTS assays, respectively), which revealed a very high antioxidant power. *S. sclarea* EO gave good results (7.79 ± 1.06 µg/mL and 2.26 ± 0.05 µg/mL) in both assays, respectively. Regarding *S. officinalis*, IC_50_ values were slightly higher (7.79 ± 1.06 µg/mL and 2.26 ± 0.05 µg/mL for DPPH and ABTS, respectively) which correspond to lower antioxidant activity. Concerning the HYs, the obtained EC_50_ values were remarkably high in the DPPH and ABTS assays which correspond to an extremely low antioxidant activity, as also obtained by expressing the values in TEAC.

Different papers reported the antioxidant activities of aromatic plant EOs due to the richness of their chemical composition [2,63,64]. *L. nobilis* EOs from different regions possess antioxidant properties [65,66,67], as well as Salvia species EOs [68].

Particularly, in our investigation oxygenated monoterpenes were the most abundant in *L. nobilis* (liquid phase) and *S. sclarea* (liquid and vapor phase) EOs, known for their high antioxidant activity [69,70,71].

## 4. Materials and Methods

### 4.1. Materials

EOs and HYs from inflorescences of *L. nobilis*, *S. officinalis* and *S. sclarea* cultivated in Tuscany (no wild site), Italy and obtained by steam distillation for 3 h (*S. officinalis* and *S. sclarea*) and 7 h (*L. nobilis*) extraction time, were directly provided by “*èssenziale*” Azienda Agricola, San Donato in Poggio (FI), Italy. Data of collection of plants: January 2020 for *L. nobilis* and June 2020 for *S. officinalis* and *S. sclarea.* All plLB Broth with Agar and Thiazolyl Blue Tetrazolium Bromide (MTT) were from Merck (Darmstadt, Germany). Gentamicin sulfate was purchased from Biochrom PAN-Bio-Tech GmbH (Aidenbach, Germany). Methanol, 2,2-Diphenyl-1-picrylhydrazyl (DPPH), 6-hydroxy-2,5,7,8-tetramethylchroman-2-carboxylic acid (Trolox), 2,2′-azinobis (3-ethylbenzothiazoline-6-sulfonic acid) diammonium salt (ABTS) and potassium persulfate (K_2_S_2_O_8_) were from Merck.

### 4.2. Gas Chromatography–Mass Spectrometry (GC–MS) Analysis

To describe the chemical composition of the EOs, a gas chromatograph with a flame ionization detector (FID) directly coupled to a mass spectrometer (MS) Perkin Elmer Clarus 500 model (Waltham, MA, USA) was used. The GC was equipped with a Varian Factor Four VF-1 capillary column. Helium was used as carrier gas at a flow rate of 1 mL/min. The injector was set to a 280 °C and the oven temperature program was as follows: from 60 °C ramped up to 220 °C at a rate of 6 °C min^−1^, and finally isothermal at 220 °C for 20 min. For liquid injections, 1 μL of each EO was diluted in 1 mL of methanol and 1 μL of the solution was injected. The Electron Impact-Mass Spectrometer (EI-MS), mass spectra were recorded at 70 eV (EI) and were scanned in the range 40–500 m/z. Ion source and the connection parts temperature was 220 °C. The GC-TIC mass spectra were obtained by the TurboMass data analysis software (Perkin Elmer). The identification of components was performed by matching their mass spectra with those stored in the Wiley and NIST 02 mass spectra libraries database. Furthermore, the linear retention indices (LRIs), (relative to C8–C30 aliphatic hydrocarbons, injected in the column at the same operating conditions described above) were calculated and compared with available retention data present in the literature. Relative percentages of all identified components were obtained by peak area normalization from GC-FID chromatograms without the use of an internal standard or correction factors and were expressed in percentages. All analyses were performed three times.

### 4.3. Head Space GC-MS Analysis

To describe the volatile fraction of the EOs and HYs vapor phase, a Perkin-Elmer Headspace Turbomatrix 40 (Waltham, MA, USA) autosampler connected to GC-MS was used for the headspace analysis. The operative conditions were performed as previously described [72,73,74]. About 1 mL of EO and 2 mL of HY were placed separately in 20 mL vials sealed with headspace PTFE-coated silicone rubber septa and caps. Quantification of identified compounds was performed by GC-FID in the same conditions described in Section 4.2.

### 4.4. Antibacterial Activities

To define the antibacterial activity of the liquid and vapor phase of *L. nobilis*, *S. officinalis* and *S. sclarea*, EOs and HYs with different methods were employed: the Minimal Inhibitory Concentration (MIC), the Minimal Bactericidal Concentration (MBC), the agar diffusion method and the Vapor Phase Test (VPT).

#### 4.4.1. Bacterial Strains and Growth Conditions

Three Gram-negative bacteria (*Escherichia coli* ATCC 25922, *Pseudomonas fluorescens* ATCC 13525 and *Acinetobacter bohemicus* DSM 102855) and two Gram-positive bacteria (*Kocuria marina* DSM 16420 and *Bacillus cereus* ATCC 10876) were used to test the antibacterial activity of *L. nobilis*, *S. officinalis* and *S. sclarea* EOs and HYs. All bacterial strains were obtained by growing cultures from the collection of the Plant Cytology and Biotechnology Laboratory (Tuscia University) grown at 26 °C (for *P. fluorescens*, *A. bohemicus* and *B. cereus*) and 37 °C (for *K. marina* and *E. coli*) in Lysogeny Broth (LB) agar.

#### 4.4.2. Microwell Dilution Method

To screen antimicrobial activities of EOs and their corresponding HYs, Minimum Inhibitory Concentration (MIC) was carried out according to the microwell dilution method in triplicate. All the matrices were diluted in LB broth (from 6.25% to 0.003%). DMSO controls, growth controls without treatments, positive controls with gentamicin (100 µg/mL to 0.049 µg/mL) and sterility controls without bacteria were added to 96 microwell plates. Bacteria at 10^6^ CFU/mL were added to each well, except to the sterility control, and were two-fold diluted. The microplates were incubated for 24 h at the corresponding growth temperature. The visualization of the bacterial growth was observed by adding 20 µL of 3-(4,5-dimethylthiazol-2-yl)-2,5-diphenyltetrazolium bromide (200 µg/mL, MTT) to each well [75,76].

Minimum Bactericidal Concentration (MBC) was carried out. Plated 10 µL of the last four dilutions from the microwell dilution method in which no bacteria growth was observed on LB agar following 24 h of incubation. The MBC value was determined by an antibacterial agent concentration for which no growth on agar was observed. The assay was carried out in triplicate. To determine the activity of the EOs and the corresponding HYs, the MBC/MIC ratio was reported. The ratio MBC/MIC > 4 reveled bacteriostatic activity, while the ratio MBC/MIC ≤ 4 revealed bactericidal activity for the tested antimicrobial agent [32].

#### 4.4.3. Agar Diffusion Method and Vapor Phase Test (VPT)

The antibacterial activities of *L. nobilis*, *S. officinalis* and *S. sclarea* EOs and HYs liquid and vapor phase were investigated, determining the diameter of the inhibition halo of the bacteria growth on agar petri dishes. Concerning the agar diffusion method, sterile disks (6 mm diameter, Oxoid), impregnated with 10 µL of samples, were placed on LB broth agar surface where the bacterial strains (approximately 10^8^ Colony Forming Unit/mL—CFU/mL) had been sown. As a positive control, 2 µL of gentamicin (10 mg/mL) was used. The petri plates were incubated for 24 h at the corresponding growth temperature, and the diameter of the inhibition halos was recorded by a vernier caliper rule [75].

The antibacterial activity of the vapor phases was evaluated by the disk volatization method. LB agar was poured into Petri plates where each bacterial suspension (10^8^ CFU/mL) was plated once it had solidified. A lower amount of agar was poured into Petri plate cover where 6 mm sterile disks had been placed and soaked with 10 µL of the EOs and the corresponding HYs. To seal the Petri plates in order to prevent any vapor leakage, liquid agar was added in the space between the cover and the base. The Petri plates were incubated for 24 h in the upside-down position at the bacterial growth temperature. The inhibition halos were measured by a vernier caliper rule. Negative controls were carried out without the essential. Agar diffusion method and VPTs were carried out in triplicate for each bacterial strain; the means and the respective standard deviations (SD) of the measured halo were reported [72,77].

### 4.5. Antioxidant Activity

#### 4.5.1. DPPH Radical Scavenging Activity Assay

To examine the radical scavenging capacity of the *L. nobilis*, *S. sclarea*, *S. officinalis* EOs and their HYs, a DPPH (2,2-diphenyl-1-picrylhydrazyl radical) assay was used [78]. The working solution was made by mixing 3.9 mg of DPPH reagent with 50 mL of methanol. The assay was carried out in 96-well plates in which wells with 100 μL of 12 geometric dilutions in methanol of each sample were added to 100 μL of DPPH solution. As controls, geometric dilutions of the samples without DPPH, Trolox geometric dilutions and blank DPPH were also measured. The plates were incubated for 30 min in dark conditions. The absorbance decreases were measured at 517 nm using a Tecan Sunrise™ UV-vis spectrophotometer. DPPH radical scavenging capacity was considered by the following relationship:

$$\%\;A.A=\left[\frac{A_{D}{-A}_{S}}{A_{D\;}}\right]\times \;100$$

where % A.A—percentage of antioxidant activity, A_D_—absorbance of blank DPPH control, A_S_—absorbance of the sample.

The IC_50_ values of the samples (mg/L) and Trolox (μM) were calculated using calibration curves by plotting the % A.A against sample concentrations. The IC_50_ parameter of the samples were reported as the required concentration to scavenge 50% of DPPH radical [79]. The activity assay was repeated three times.

TEAC value, which correlates to the concentration of Trolox with the dry weigh of the tested sample, was calculated from the Trolox IC_50_ (µM) and the sample IC_50_ (mg/L):

$$\mathrm{TEAC}=\;\frac{{\mathrm{IC}50}_{\mathrm{trolox}}}{{\mathrm{IC}50}_{\mathrm{sample}}}$$

#### 4.5.2. ABTS Radical Scavenging Activity Assay

The radical activities of the *L. nobilis*, *S. sclarea* and *S. officinalis* EOs and their HYs were also calculated using the pre-formed radical monocation of 2,2′-azinobis-(3-ethylbenzothiazoline-6-sulfonic acid) (ABTS+•), as reported by Pellegrini et al. [80], with some variations. The working aqueous solution was made by mixing potassium persulfate (140 mM) with ABTS reagent (7 mM). The solution was kept for 16 h at room temperature in the dark. Before use, the ABTS+• solution was diluted in ethanol 100% to give an absorbance of 0.70 ± 0.02 at 734 nm. In total, 20 μL of each sample was added to 1980 μL of working solution; as a control, 20 μL of ethanol were used. Therefore, the obtained solutions were incubated for 5 min at room temperature and the absorbances were measured at 734 nm by a Jasco V-630 UV-Visible spectrophotometer and operating Spectra Manager™ software. The final values expressed as a percentage of inhibition against samples concentrations were likened against a standard curve of Trolox [81]. The concentrations used for Trolox range from 0.15625 μM to 2.5 μM.

### 4.6. Statistical Analysis

The results were expressed as means ± standard deviation (SD) and the ANOVA test (one-way analysis of variance test) using GraphPad Prism software (GraphPad Prism 5.0, GraphPad Software Inc., San Diego, CA, USA) was used to evaluate statistical discrepancies between the groups (*p* values < 0.05).

## 5. Conclusions

To the best of our knowledge, this is the first work reporting the liquid and vapor phase chemical composition of *L. nobilis*, *S. sclarea* and *S. officinalis* EOs from the Tuscany region (Italy) investigated by GC-MS and HS-GC/MS techniques to describe the volatile profile of the respective HYs by HS-GC/MS. The antibacterial activity was also evaluated by microdilution and the disc diffusion method, as well as the antioxidant activity by DPPH and ABTS assays. The tested EOs exhibited antibacterial and antioxidant activities, particularly *L. nobilis* vapor phase, which determined the major inhibition halo. HYs showed to not be antibacterial at the tested concentrations, while the antioxidant properties were extremely low compared to that of the EOs. The obtained results show the potential use of EOs as possible natural antibacterial substances and sources of bioactive molecules. To assess HY biological properties further, tests will be run on other environmental bacterial strains. From a general point of view, our findings confirm the importance of bioactive plant molecules, and as a consequence it will be important to maintain productivity and stability of cultivated plants. In this view, in situ and ex situ conservation of the progenitors of domesticated plants, crop-wild relatives, become necessary, and the gene bank to maintain biodiversity should be improved [82,83,84].

## Figures and Tables

**Table 1:** Chemical composition (%) of liquid and vapor phase *L. nobilis* essential oils (EO).

N°	Component ^1^	LRI ^2^	LRI ^3^	*L. nobilis* (%) ^4^	*L. nobilis* (%) ^5^
1	α-pinene	941	943	16.7	39.0
2	camphene	945	946	1.4	2.6
3	β-myrcene	981	983	3.4	3.4
4	β-pinene	987	986	13.6	10.9
5	α-phellandrene	994	996	0.7	1.1
6	α-terpinene	1005	1008	1.0	2.0
7	o-cymene	1020	1021	0.2	0.9
8	limonene	1026	1023	1.2	2.6
9	1,8-cineole	1028	1027	42.2	33.5
10	β-ocimene	1038	1037	1.2	0.4
11	γ-terpinene	1051	1054	2.1	2.8
12	terpinolene	1075	1078	0.6	0.1
13	linalool	1090	1092	3.1	0.6
14	terpinen-4-ol	1158	1160	0.8	0.1
15	linalyl acetate	1249	1252	0.2	-
16	bornyl acetate	1271	1275	0.3	-
17	eugenol	1341	1345	1.6	-
18	α-terpinyl acetate	1350	1353	7.5	-
19	α-copaene	1375	1379	0.1	-
20	β-elemene	1388	1391	0.5	-
21	β-caryophyllene	1422	1426	0.8	-
22	γ-muurolene	1500	1501	0.2	-
23	α-farnesene	1504	1506	0.1	-
24	δ-cadinene	1533	1530	0.2	-
25	spathulenol	1577	1581	0.1	-
26	caryophyllene oxide	1580	1583	0.1	-
	SUM (%)			99.9	100.0
	Monoterpene hydrocarbons			42.1	65.8
	Oxygenated monoterpenes			54.1	34.2
	Sesquiterpene hydrocarbons			1.9	-
	Oxygenated sesquiterpenes			0.2	-
	Others			1.6	-

^1^ N°: the components are reported according to their elution order on apolar column; ^2^ LRI: Linear Retention indices measured on apolar column; ^3^ Linear Retention indices from literature; ^4^ Percentage mean values of *L. nobilis* EO components (%); ^5^ Percentage mean values of *L. nobilis* EO components (vapor phase)—not detected.

**Table 2:** Chemical composition (%) of liquid and vapor phase *S. officinalis* EO.

N°	Component ^1^	LRI ^2^	LRI ^3^	*S. officinalis* (%) ^4^	*S. officinalis* (%) ^5^
1	α-pinene	941	943	6.0	19.7
2	camphene	945	946	7.9	2.2
3	β-pinene	987	986	3.8	5.9
4	p-cymene	1015	1016	1.1	1.3
5	1,8-cineole	1028	1027	30.4	48.4
6	γ-terpinene	1051	1054	0.3	2.3
7	α-thujone	1095	1097.4	9.7	7.0
8	chrysanthenone	1102	1100	6.8	3.9
9	camphor	1123	1126	17.1	8.7
10	borneol	1148	1152	1.6	0.3
11	α-terpineol	1180	1183	0.3	-
12	bornyl acetate	1271	1275	1.1	-
13	thymol	1282	1279.9	0.1	-
14	4-terpinenyl acetate	1287	1286	0.6	0.1
15	α-gurjunene	1418	1420	0.7	-
16	β-caryophyllene	1422	1426	3.6	0.1
17	humulene	1471	1473	2.5	-
18	γ-gurjunene	1477	1479	3.9	-
19	γ-muurolene	1500	1501	0.2	-
20	guaia -1(10),11-diene	1505	1508	1.0	-
21	δ-cadinene	1533	1530	0.2	-
22	viridiflorol	1571	1580	0.6	-
23	caryophyllene oxide	1580	1583	0.5	-
	SUM (%)			100.0	99.9
	Monoterpene hydrocarbons			19.1	31.4
	Oxygenated monoterpenes			67.7	68.4
	Sesquiterpene hydrocarbons			11.1	0.1
	Oxygenated sesquiterpenes			1.1	-
	Others			1.0	-

^1^ The components are reported according to their elution order on apolar column; ^2^ Linear Retention indices measured on apolar column; ^3^ Linear Retention indices from literature; ^4^ Percentage mean values of *S. officinalis* EO components (%); ^5^ Percentage mean values of *S. officinalis* EO components (vapor phase)—not detected.

**Table 3:** Chemical composition (%) of liquid and vapor phase *S. sclarea* EO.

N°	Component ^1^	LRI ^2^	LRI ^3^	*S. sclarea* (%) ^4^	*S. sclarea* (%) ^5^
1	β-pinene	987	986	3.1	15.2
2	limonene	1026	1023	0.6	2.5
3	cis-β-ocimene	1033	1032	1.5	7.7
4	trans-β-ocimene	1041	1043	0.8	4.1
5	linalool	1090	1092	11.1	28.9
6	α-terpineol	1180	1183	1.5	1.3
7	linalyl acetate	1249	1252	62.6	30.1
8	geranyl acetate	1364	1366	1.4	2.6
9	α-copaene	1375	1379	1.8	0.8
10	β-cubebene	1388	1390	2.7	5.0
11	β-caryophyllene	1422	1426	3.4	0.8
12	β-copaene	1442	1445	6.7	0.9
13	γ-gurjunene	1477	1479	0.7	-
14	γ-muurolene	1500	1501	1.1	0.1
15	δ-cadinene	1533	1530	1.0	-
	SUM (%)			100.0	100.0
	Monoterpene hydrocarbons			6.0	29.5
	Oxygenated monoterpenes			76.6	62.9
	Sesquiterpene hydrocarbons			17.4	7.6
	Oxygenated sesquiterpenes			-	-
	Others			-	-

^1^ The components are reported according to their elution order on apolar column; ^2^ Linear Retention indices measured on apolar column; ^3^ Linear Retention indices from literature; ^4^ Percentage mean values of *S. sclarea* EO components (%); ^5^ Percentage mean values of *S. sclarea* EO components (vapor phase)—not detected.

**Table 4:** Chemical composition (%) of vapor phase *L. nobilis*, *S. officinalis* and *S. sclarea* hydrolates (HYs).

N°	Component ^1^	LRI ^2^	LRI ^3^	*L. nobilis* (%) ^4^	*S. officinalis* (%) ^5^	*S. sclarea* (%) ^6^
1	1,8-cineole	1028	1027	65.1	61.4	-
2	β-terpinene	1035	1036	-	2.3	-
3	β-ocimene	1038	1037	0.6	-	-
4	linalool	1090	1092	-	-	89.5
5	α-thujone	1095	1097.4	11.1	8.4	-
6	β-thujone	1106	1108.3	4.7	3.4	-
7	camphor	1123	1126	9.1	22.5	-
8	borneol	1148	1152	8.4	-	-
9	α-terpineol	1180	1183	-	-	10.5
10	bornyl acetate	1271	1275	-	1.4	-
11	α-terpinyl acetate	1350	1353	1.0	0.6	-
	SUM			100.0	100.0	100.0
	Monoterpene hydrocarbons			0.6	2.3	-
	Oxygenated monoterpenes			99.4	97.7	100.0
	Sesquiterpene hydrocarbons			-	-	-
	Oxygenated sesquiterpenes			-	-	-
	Others			-	-	-

^1^ The components are reported according to their elution order on apolar column; ^2^ Linear Retention indices measured on apolar column; ^3^ Linear Retention indices from literature; ^4^ Percentage mean values of *L. nobilis* HY components (%); ^5^ Percentage mean values of *S. officinalis* HY components; ^6^ Percentage mean values of *S. sclarea* HY components—not detected.

**Table 5:** Antibacterial activities for EO and HY of *L. nobilis.*

	*L. nobilis* EO			*L. nobilis* HY		*L. nobilis* EO		*L. nobilis* HY	
Strains	MIC ^1^	MBC ^2^	MBC/MIC Ratio	MIC ^1^	MBC ^2^	IZ ^3^	VIZ ^4^	IZ ^3^	VIZ ^4^
*E. coli*	3.13	3.13	1.00	na	na	18.67 ± 2,31	-	-	-
*P. fluorescens*	3.13	3.13	1.00	na	na	7.33 ± 0.58	-	-	-
*A. bohemicus*	0.78	1.56	0.50	na	na	17.67 ± 2.31	45.67 ± 4.04	-	-
*K. marina*	1.56	6.25	0.25	na	na	24.67 ± 3.21	26.67 ± 2.52	-	-
*B. cereus*	1.56	1.56	1.00	na	na	37.67 ± 2.08	47.33 ± 2.52	-	-

^1^ MIC: Minimal inhibitory concentration expressed in % of EO and HY treatments; ^2^ MBC: Minimal bactericidal concentration expressed in % of EO and HY treatments; ^3^ IZ^:^ growth inhibition zone by disc diffusion assay expressed in mm; ^4^ VIZ: growth inhibition zone by vapor phase test expressed in mm; na: not attained—not detected; Values are expressed as means ± SD. *p* < 0.05 from one-way analysis of variance test (ANOVA).

**Table 6:** Antibacterial activities for EO and HY of *S. officinalis.*

	*S. officinalis* EO			*S. officinalis* HY		*S. officinalis* EO		*S. officinalis* HY	
Strains	MIC ^1^	MBC ^2^	MBC/MIC Ratio	MIC ^1^	MBC ^2^	IZ ^3^	VIZ ^4^	IZ ^3^	VIZ ^4^
*E. coli*	6.25	6.25	1	na	na	16.67 ± 1.53	-	-	-
*P. fluorescens*	6.25	6.25	1	na	na	8.00 ± 1.00	-	-	-
*A. bohemicus*	0.39	0.78	0.50	na	na	13.67 ± 1.53	21.67 ± 1.53	-	-
*K. marina*	1.56	1.56	1	na	na	38.33 ± 2.89	26.67 ± 1.15	-	-
*B. cereus*	0.78	0.78	1	na	na	24.33 ± 3.06	23.00 ± 3.61	-	-

^1^ Minimal inhibitory concentration expressed in % of EO and HY treatments; ^2^ Minimal bactericidal concentration expressed in % of EO and HY treatments; ^3^ Growth inhibition zone by disc diffusion assay expressed in mm; ^4^ Growth inhibition zone by vapor phase test expressed in mm; na: not attained—not detected. Values are expressed as means ± SD. *p* < 0.05 from one-way analysis of variance test (ANOVA).

**Table 7:** Antibacterial activities for EO and HY of *S. sclarea.*

	*S. sclarea* EO			*S. sclarea* HY		*S. sclarea* EO		*S. sclarea* HY	
Strains	MIC ^1^	MBC ^2^	MBC/MIC Ratio	MIC ^1^	MBC ^2^	IZ ^3^	VIZ ^4^	IZ ^3^	VIZ ^4^
*E. coli*	12.50	12.50	1	na	na	16.67 ± 1.53	-	-	-
*P. fluorescens*	na	na	-	na	na	8.00 ± 1.00	-	-	-
*A. bohemicus*	1.56	1.56	1	na	na	12.67 ± 2.52	-	-	-
*K. marina*	6.25	6.25	1	na	na	18.67 ± 0.58	-	-	-
*B. cereus*	6.25	6.25	1	na	na	10.67 ± 1.15	-	-	-

^1^ Minimal inhibitory concentration expressed in % of EO and HY treatments; ^2^ Minimal bactericidal concentration expressed in % of EO and HY treatments; ^3^ Growth inhibition zone by disc diffusion assay expressed in mm; ^4^ Growth inhibition zone by vapor phase test expressed in mm; na: not attained—not detected. Values are expressed as means ± SD. *p* < 0.05 from one-way analysis of variance test (ANOVA).

**Table 8:** Effects of EO and HY of *L. nobilis* in DPPH and ABTS antioxidant assays.

		*L. nobilis* EO	*L. nobilis* HY
DPPH	IC_50_ *	0.18 ± 0.04	218.10 ± 29.60
	TEAC **	92.97 ± 6.76	0.08 ± 0.01
ABTS	IC_50_ *	2.58 ± 0.08	391.38 ± 8.72
	TEAC **	158.49 ± 5.15	1.19 ± 0.21

* µg/mL of essential oil; ** µM of Trolox equivalent/mg of essential oil. IC_50_: the half maximal inhibitory concentration; TEAC: Trolox equivalent antioxidant capacity. Values are expressed as means ± SD. *p* < 0.05.

**Table 9:** Effects of EO and HY of *S. officinalis* in DPPH and ABTS antioxidant assays.

		*S. officinalis* EO	*S. officinalis* HY
DPPH	IC_50_ *	14.10 ± 0.17	135.58 ± 33.32
	TEAC **	1.28 ± 0.00	0.14 ± 0.03
ABTS	IC_50_ *	43.64 ± 2.51	551.38 ± 17.33
	TEAC **	9.26 ± 0.55	0.76 ± 0.02

* µg/mL of essential oil; ** µM of Trolox equivalent/mg of essential oil. Values are expressed as means ± SD. *p* < 0.05.

**Table 10:** Effects of EO and HY of *S. sclarea* in DPPH and ABTS antioxidant assays.

		*S. sclarea* EO	*S. sclarea* HY
DPPH	IC_50_ *	7.79 ± 1.06	200.43 ± 28.46
	TEAC **	2.34 ± 0.36	0.09 ± 0.01
ABTS	IC_50_ *	2.26 ± 0.05	479.27 ± 7.89
	TEAC **	186.23 ± 4.30	0.87 ± 0.01

* µg/mL of essential oil; ** µM of Trolox equivalent/mg of essential oil. Values are expressed as means ± SD. *p* < 0.05.
